# Comparative proteomic and metabolomic profiling of citrus fruit with enhancement of disease resistance by postharvest heat treatment

**DOI:** 10.1186/1471-2229-13-44

**Published:** 2013-03-16

**Authors:** Ze Yun, Huijun Gao, Ping Liu, Shuzhen Liu, Tao Luo, Shuai Jin, Qiang Xu, Juan Xu, Yunjiang Cheng, Xiuxin Deng

**Affiliations:** 1 Key Laboratory of Horticultural Plant Biology of Ministry of Education, Huazhong Agricultural University, Wuhan, Hubei, 430070, P.R. China

**Keywords:** Disease resistance, H_2_O_2_, Heat treatment, Lignin postharvest storage, Metabolomics, Proteomics

## Abstract

**Background:**

From field harvest to the consumer’s table, fresh citrus fruit spends a considerable amount of time in shipment and storage. During these processes, physiological disorders and pathological diseases are the main causes of fruit loss. Heat treatment (HT) has been widely used to maintain fruit quality during postharvest storage; however, limited molecular information related to this treatment is currently available at a systemic biological level.

**Results:**

Mature ‘Kamei’ Satsuma mandarin (*Citrus unshiu* Marc.) fruits were selected for exploring the disease resistance mechanisms induced by HT during postharvest storage. Proteomic analyses based on two-dimensional gel electrophoresis (2-DE), and metabolomic research based on gas chromatography coupled to mass spectrometry (GC-MS), and liquid chromatography quadrupole time-of-flight mass spectrometry (LC-QToF-MS) were conducted. The results show resistance associated proteins were up-regulated in heat treated pericarp, such as beta-1, 3-glucanase, Class III chitinase, 17.7 kDa heat shock protein and low molecular weight heat-shock protein. Also, redox metabolism enzymes were down-regulated in heat treated pericarp, including isoflavone reductase, oxidoreductase and superoxide dismutase. Primary metabolic profiling revealed organic acids and amino acids were down-regulated in heat treated pericarp; but significant accumulation of metabolites, including tetradecanoic acid, oleic acid, ornithine, 2-keto-d-gluconic acid, succinic acid, turanose, sucrose, galactose, myo-inositol, glucose and fructose were detected. Noticeably, H_2_O_2_ content decreased, while, lignin content increased in heat treated pericarp compared to the control, which might increase fruit resistibility in response to external stress. Also, flavonoids, substances which are well-known to be effective in reducing external stress, were up-regulated in heat treated pericarp.

**Conclusions:**

This study provides a broad picture of differential accumulation of proteins and metabolites in postharvest citrus fruit, and gives new insights into HT improved fruit disease resistance during subsequent storage of ‘Kamei’ Satsuma mandarin. Interpretation of the data for the proteins and metabolites revealed reactive oxygen species (ROS) and lignin play important roles in heat treatment induced fruit resistance to pathogens and physiological disorders.

## Background

Fresh citrus fruits, from harvest to human consumption, require a lengthy period for shipping, storing and marketing. During that time, the main damage to fruit comes from biological diseases and physical damage. Storage pre-treatments, such as biotic or abiotic typological treatments, can be used to inhibit disease and extend the shelf-life. Among the various types of treatment available, heat treatment has previously been reported to induce disease resistance in apples, citrus, grapes, peaches, pears and mangos against a multitude of pathogens [1-3].

In the fruit industry, hot water treatment is an alternative physical treatment, which has been used in citrus fruit storage since 1922 [4]. More recently, hot air and hot water systems have been widely adopted in several countries to inhibit postharvest decay of fruits and to alleviate chilling injuries during storage. Heat treatment (HT) has the advantage of minimizing surface infection in pericarp without leaving any chemical residues [5]. HT can prevent the development of plant pathogens and disease by heat-killing disease organisms or by inhibiting pathogen mycelial growth, by removing surface insects and by structurally altering epicuticular wax [6]. HT can also enhance fruit resistance to chilling injury in cold-sensitive cultivars, and help the fruit retain quality during cold storage and help maintain shelf life [7]. Aside from controlling pathogenic organisms, HT may also reduce disease by changing the physical characteristics of the interior pericarp. For example, HT not only delayed fruit ripening and coloration, inhibited fruit softening, and slowed ethylene production [3], it also increased fruit hardness, as well as increasing the content of polyphenolics, flavonoids and dopamine [6-8]. However, less is understood about the regulatory genes which are active in the response to microbial attack, and there is no exhaustive metabolomics profile detailing the changes in heat treated pericarp.

In the past few decades, researchers have given increasing attention to the study of gene expression in heat treated fruits. Sapinitskaya *et al.*[9] reported HT activates stress genes and lipid modification genes in response to chilling stress in grapefruit, such as glutathione-dependent formaldehyde dehydrogenase, dehydrin, heat shock protein (HSP), ascorbate peroxidase, superoxide dismutase, fatty acid desaturase and lipid transfer protein. Some scientists discovered HT induces phenylalanine ammonia lyase and accumulates phenylpropanoid compounds, which play a vital role in heat-induced fruit resistibility [2]. Dixon e*t al.*[10] studied the functions of phenylpropanoid compounds in plants involved in local and systemic signalling for defence-gene induction. However, little information is available related to which particular class of phenylpropanoid compounds plays a core role in defensive functions, and even less is known about the regulatory genes which orchestrate the rapid induction of phenylpropanoid defences in response to HT. Proteome analysis reveals the induction of HSPs, allergen proteins, dehydrin, and other proteins involved in the stress response. Repression of polyphenol oxidase caused by HT may constitute the molecular basis for the protection against chilling stress in peach fruit [3]. However, few proteins involved in HT were reported to have induced fruit resistance to deterioration, especially in citrus fruit.

Also, proteomics analysis and metabolic profiling approaches make possible comparative parallel analyses of global changes at the proteome and metabolome levels, facilitating an understanding of the relationships between changes in specific proteins and subsequent alterations in metabolites in response to HT. In the present study, fruits of ‘Kamei’ Satsuma mandarin (*Citrus unshiu* Marc.) received HT. The comparative proteomics analysis and metabolic profiling (the primary metabolome and the secondary metabolome) analysis were performed based on two-dimensional electrophoresis (2-DE), gas chromatography and mass spectrometry (GC-MS) and high-performance liquid chromatography/electrospray ionization-time of flight-mass spectrometry (HPLC-QTOF-MS). Comprehensive physiological and biochemical analysis were completed. Results will provide additional theoretical evidence to improvement of disease resistance in heat treated pericarp.

## Results

Satsuma mandarin is one of the main citrus crops in East Asia, including China, South Korea and Japan. However, this cultivar has poor storability mainly because it is susceptible to biotic stress and physical damage. HT improved fruit resistance to stress and damage effectively and inhibited postharvest decay of fruits. In present study, HT fruits were dipped in a 52°C warm water bath for 2 min, and control fruits were dipped in a 25°C water bath for 2 min. After treatments, fruits were stored in a ventilated warehouse (storage temperature: 12–16°C; relative humidity: 90–95%). Samples were collected at 2 h, and 1, 2, 3, 4, 6, 9, 12, 16, 20, 24, 28, 32, 38, 44, 50 and 60 days after treatments (DAT) for further analysis.

### HT increased fruit weight loss slightly, but did not affect fruit quality

**Figure 1:**
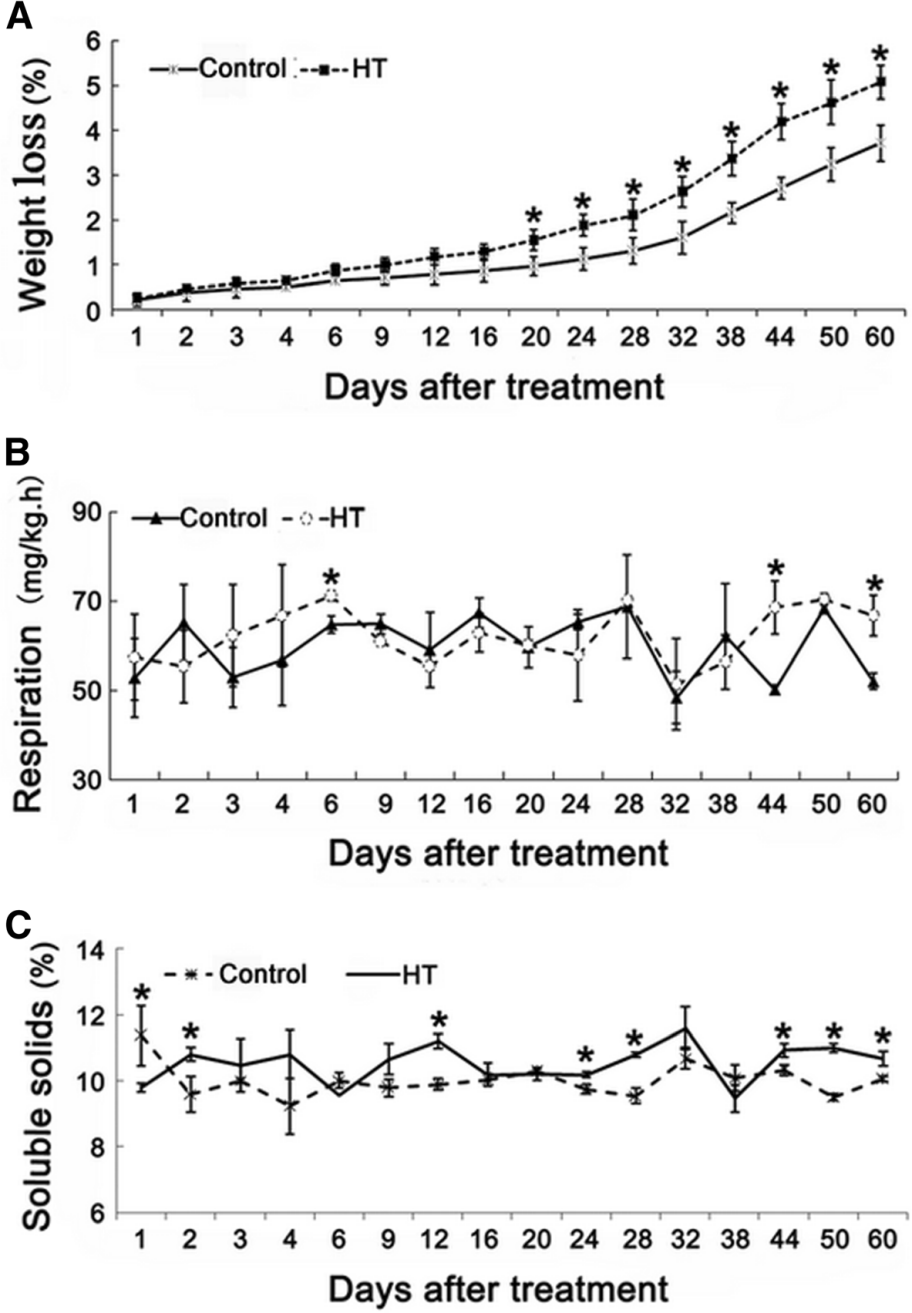
**The influence of heat treatment on ‘Kamei’ Satsuma mandarin fruit quality during the entire period of storage at ambient temperature.** Parameters related to fruit quality, including weight loss, respiration rate, and soluble solids content, were evaluated. 30 fruits (each sample) and three replications were applied to quality analysis. Control: fruits dipped in a water bath at 25°C for 2 min; HT: fruits dipped in a water bath at 52°C for 2 min. An analysis of statistically significant differences was conducted between HT and control pericarp at the same period using Student’s t-test. *: significant difference (*P* < 0.05). Mean values and SE bars are provided.

Fruit quality directly determines its commercial value. Parameters related to fruit quality, including weight loss, respiration rate, and soluble solids content, were evaluated to investigate the influence of HT on fruit quality. Weight loss increased significantly in heat treated pericarp compared to control pericarp during the storage period (Figure 1A). However, no obvious changes were observed in the respiration rate and soluble solids content between HT and control fruits during the entire storage period (Figures 1B, C). Since soluble solids content is a well-known standard in measuring fruit internal quality and respiration acts as an important standard for fruit storability, the minor changes in soluble solids content and respiration indicated that heat treatment did not affect fruit flesh quality.

### HT suppression of *Penicillium* germination on pericarp

**Figure 2:**
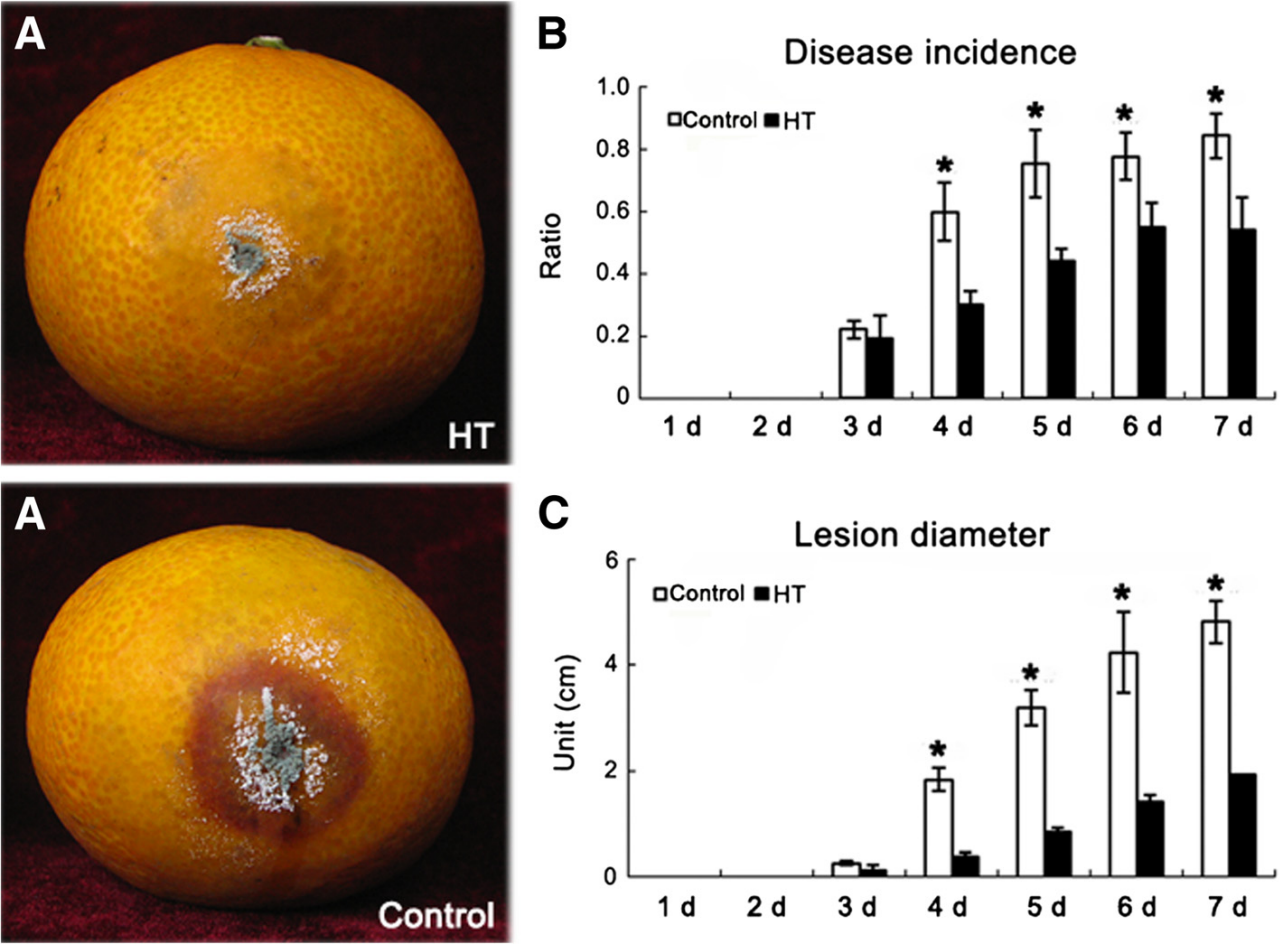
**Effects of HT on disease development in ‘Kamei’ Satsuma mandarin fruit during storage at ambient temperature.** 150 fruits were used for fungal infection. A uniform lesion (4 mm deep, 3 mm wide) was made at the equator of the fruit using a sterile nail. Aliquots of 20 μl suspension of *Penicillium italicum* at 1×10^5^ spores ml^–1^ were inoculated into each wound site. After fungal inoculation, the incidence of disease in fruit was detected at 1, 2, 3, 4, 5, 6 and 7 days after *Penicillium* inoculation. Statistically significant differences analysis was conducted between HT and control pericarp during the same period using Student’s t-test. *: significant difference (*P* < 0.05). Mean values and SE bars are provided. **A**: Fruit status at 3 d after *Penicillium* inoculation; **B**: Disease incidence ratio; **C**: Fruit lesion diameter.

Artificial inoculation tests were conducted with the blue mould *Penicillium italicum* to assess the effects of HT on the fruit’s response to biotic stresses. Few spores had germinated on the peel of HT fruits 4 d after inoculation, but about 90% of the control fruits showed typical symptoms of blue mould development at the inoculation sites (Figure 2A). As for the rate of disease incidence, the incidence of disease on control fruit was significantly higher than on HT fruit from 4–7 d after inoculation (Figure 2B); lesion diameters were significantly higher in control fruit than HT fruit from 4–7 d after inoculation (Figure 2C). Based on the above experiments, HT effectively inhibited the germination of *Penicillium* on pericarp.

### Differentially accumulated proteins in HT citrus pericarp

**Figure 3:**
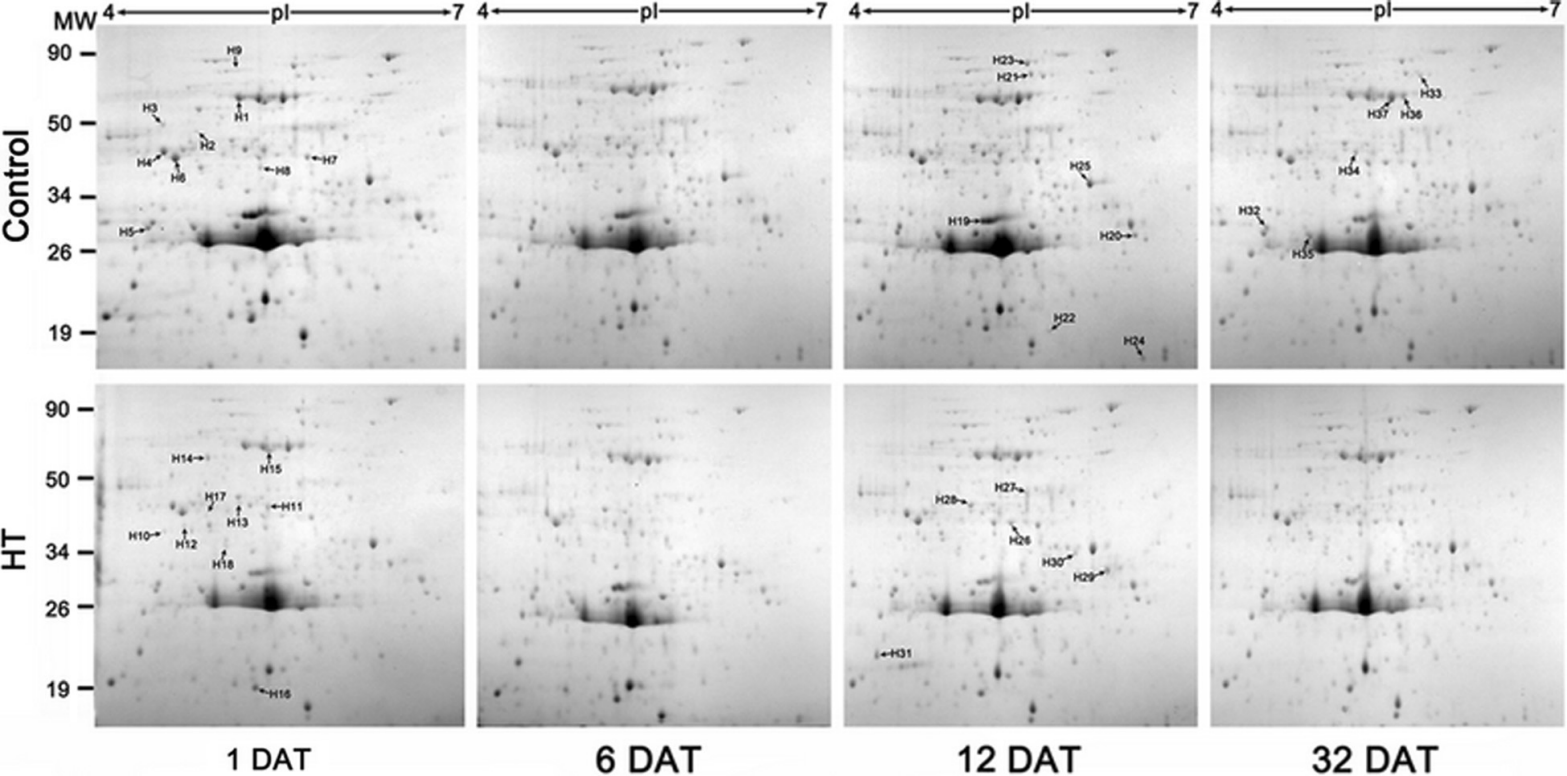
**Representative 2-DE profiles of proteins from different treated Satsuma mandarin fruits during postharvest storage at ambient temperature.** Total proteins were extracted from the fruit at 1, 6, 12 and 32 DAT. Proteins (l mg) were separated in the first dimension on IPG strip (17 cm, pH 4–7) and in the second dimension on a 15% SDS-PAGE gel. More than 600 spots were observed. 37 of 50 differential accumulated proteins were identified using MALDI TOF/TOF. The proteins were classified into 5 categories, including glycolysis and TCA cycle (9 proteins, H1-9), redox reactions (9 proteins, H10-18), stress response (7 proteins, H19-25), other metabolism (6 proteins, H26-31), protein folding (6 proteins, H32-37). Table one shows detailed information related to the proteins.

To explore the differential expression of proteins between HT and control at the same period, the pericarp of ‘Kamei’ Satsuma mandarin fruit were analysed with samples collected at 1, 6, 12 and 32 DAT. After 2-DE analysing, more than 600 protein spots were reproducibly detected (Figure 3). The protein relative abundance was obtained with the PDQuest 2-D software version 7.4. Protein spots were scored only when they were reproducibly observed in four independent replicates. Data were normalized as a percentage of the total density of all spots on the corresponding gel. Comparative analysis was carried out using 1.5-fold comparative analysis and one-way analysis of variance (ANOVA). Result showed there were only 50 differentially displayed protein spots whose abundance was altered at least 1.5-fold when comparing the HT and control during the same period of storage. Of these, 37 were identified using Applied Biosystems 4800 matrix-assisted laser desorption/ionization-time-of-flight tandem mass spectrometry (MALDI-TOF MS) based on tryptic peptide sequences (Figure 3, Table 1). Based on previously reported functions of these proteins, they were classified into five categories (Table 1): glycolysis and TCA cycle (nine proteins, 24%), redox regulation (nine proteins, 24%), stress response (seven proteins, 19%), other metabolism (six proteins, 16%), and protein folding (six proteins, 16%).

**Table 1:** Identities of HT induced/decreased proteins in the peels of Satsuma mandarin fruit

**Glycolysis and TCA cycle: 9**								
**No.**	**Protein name**	**Protein accumulation**	**Source organism**	**GI no.**	**TheoMr/pI**	**Expt Mr/pI**	**PC**	**MS**
H1	Enolase		*Glycine max*	42521309	47.97/5.31	50.84/5.23	8	443
H2	Phosphoglycerate kinase, putative		*Arabidopsis thaliana*	15223484	50.02/8.27	41.88/4.90	9	108
H3	Putative 2-oxoglutarate dehydrogenase E2 subunit		*Oryza sativa*	48716382	49.46/6.78	43.64/4.62	4	105
H4	Malate dehydrogenase, cytoplasmic		*Beta vulgaris*	11133601	35.81/5.89	37.61/4.61	7	347
H5	Triosephosphate isomerase, cytosolic (TIM)		*Zea mays*	136063	27.23/5.52	24.97/4.47	9	310
H6	Malate dehydrogenase, mitochondrial precursor		*Citrullus lanatus*	126896	36.40/8.88	36.36/4.71	8	376
H7	Pyruvate dehydrogenase E1 beta subunit isoform 2		*Zea mays*	162458637	40.23/5.56	35.91/5.72	5	302
H8	NAD-malate dehydrogenase		*Nicotiana tabacum*	5123836	43.67/8.03	34.10/5.38	11	436
H9	Putative2,3-bisphosphoglycerate- independent phosphoglycerate mutase		*Arabidopsis thaliana*	15982735	60.67/5.27	61.18/5.12	9	207
**Redox regulation: 9**								
**No.**	**Protein name**	**Protein accumulation**	**Source organism**	**GI no.**	**TheoMr/pI**	**Expt Mr/pI**	**PC**	**MS**
H10	Isoflavone reductase related protein		*Pyrus communis*	3243234	33.80/6.02	33.12/4.53	5	92
H11	Oxidoreductase, zinc-binding dehydrogenase family protein		*Arabidopsis thaliana*	15220854	41.13/8.46	37.58/5.41	6	93
H12	Putative NAD(P)H oxidoreductase, isoflavone reductase		*Arabidopsis thaliana*	19310585	34.24/6.61	34.43/4.71	4	67
H13	Aldo/keto reductase family protein		*Arabidopsis thaliana*	42571931	27.76/6.33	38.36/5.16	6	164
H14	2-oxoacid dehydrogenase family protein		*Arabidopsis thaliana*	15240454	50.27/9.19	47.44/4.92	7	168
H15	Mitochondrial processing peptidase		*Solanum tuberosum*	587562	54.62/5.99	49.29/5.42	4	65
H16	Copper/zinc superoxide dismutase		*Citrus limon*	33340236	15.20/5.46	17.02/5.27	4	263
H17	Putative NAD(P)H oxidoreductase, isoflavone reductase		*Arabidopsis thaliana*	19310585	34.24/6.61	34.47/4.92	3	85
H18	Chloroplast stromal ascorbate peroxidase		*Vigna unguiculata*	45268439	39.95/7.06	31.00/5.06	9	191
**Stress response: 7**								
**No.**	**Protein name**	**Protein accumulation**	**Source organism**	**GI no.**	**TheoMr/pI**	**Expt Mr/pI**	**PC**	**MS**
H19	Abscisic stress ripening-like protein		*Prunus persica*	16588758	20.74/5.68	29.89/5.25	4	316
H20	Class III chitinase		*Benincasa hispida*	5919201	33.03/9.8	26.84/6.32	3	65
H21	Heat shock protein 60		*Prunus dulcis*	24637539	58.06/5.26	88.79/5.66	10	205
H22	17.7 kDa heat shock protein		*Carica papaya*	37933812	17.77/6.39	19.12/5.95	2	98
H23	Putative heat shock 70 KD protein, mitochondrial precursor		*Oryza sativa*	27476086	70.68/5.45	92.99/5.65	17	358
H24	Low molecular weight heat-shock protein		*Pseudotsuga menziesii*	1213116	18.18/5.82	17.78/6.39	4	122
H25	Beta-1, 3-glucanase		*Citrus sinensis*	2274915	37.32/9.19	34.38/6.18	7	98
**Other metabolism: 6**								
**No.**	**Protein name**	**Protein accumulation**	**Source organism**	**GI no.**	**TheoMr/pI**	**Expt Mr/pI**	**PC**	**MS**
H26	Fiber annexin		*Gossypium hirsutum*	3493172	36.20/6.34	36.23/5.53	5	70
H27	Alpha-amylase		*Citrus reticulata*	20336385	17.05/4.91	44.16/5.64	8	177
H28	Glutamine synthetase		*Elaeagnus umbellata*	47933888	39.33/6.10	39.38/5.16	5	134
H29	Putative inorganic pyrophosphatase		*Oryza sativa*	46805453	20.45/4.61	28.58/6.31	4	74
H30	Thiazole biosynthetic enzyme, chloroplast precursor		*Citrus sinensis*	6094476	37.74/5.40	31.35/6.04	12	447
H31	Lipocalin protein		*Capsicum annuum*	50236424	21.41/7.66	19.0/4.33	3	143
**Protein folding: 6**								
**No.**	**Protein name**	**Protein accumulation**	**Source organism**	**GI no.**	**TheoMr/pI**	**Expt Mr/pI**	**PC**	**MS**
H32	Catalytic/coenzyme binding		*Arabidopsis thaliana*	18404496	34.97/8.37	25.54/4.50	6	252
H33	Chaperonin-60 beta subunit		*Solanum tuberosum*	1762130	63.26/5.72	57.6/5.77	12	283
H34	Protein disulfide isomerase		*Zea mays]*	162461925	40.43/6.29	37.7/5.26	9	165
H35	Transcription factor APFI		*Arabidopsis thaliana*	13507025	30.14/6.24	28.15/5.14	3	89
H36	Putative ATP synthase beta subunit		*Oryza sativa*	56784991	45.93/5.33	51.13/5.66	20	695
H37	Putative ATP synthase beta subunit		*Oryza sativa*	56784991	45.93/5.33	50.0/5.6	20	808

Samples at 1, 6, 12 and 32 DAT were used for differential analysis of proteins accumulation. After 2-DE, protein spot intensities were quantified using PDQuest 2-D software version 7.4. Comparative and statistical analysis shows there were only 50 differentially displayed protein spots whose abundance was altered at least 1.5-fold between the HT and control during the same time period. Of these, 34 proteins were successfully identified using Applied Biosystems 4800 matrix-assisted laser desorption/ionization-time-of-flight tandem mass spectrometry (MALDI-TOF MS) analysis. Protein relative accumulation is represented by the column configuration, and accumulation at 1, 6, 16 and 32 DAT is shown from left to right. Protein name and GI No. are from the National Center for Biotechnology Information (NCBI) database (http://www.ncbi.nlm.n ih.gov/guide/). Experimental molecular mass and pI (Exp. M/pI) were calculated using PDQuest software (version 7.4), and mass is shown in kDa. PC: Peptide count. MS: Mascot score.

### Differentially accumulated metabolites in HT citrus pericarp

Differentially accumulated metabolites were studied to investigate the changes in metabolic composition between HT and control pericarp. The biochemical changes were determined by GC-MS and HPLC-QTOF-MS. A parallel analysis of non-polar and polar extracts using methanol extraction enabled the detection of a large number of compounds of different classes.

A total of 62 identified metabolites were monitored in the same sample sets using GC-MS, including mainly alcohols, amino acids, sugars, organic acids, and fatty acids. After conducting an analysis of significance with ANOVA (*P* <0.05), 45 detected metabolites, mostly organic acids, sugars and amino acids, were found to be significantly up/down-regulated in heat treated pericarp when compared to control pericarp with the same storage period (Table 2).

**Table 2:** Differential accumulated primary metabolites in heat treated pericarp compared to control pericarp

**Sugars**	**2 h**	**1 d**	**6 d**	**32 d**
Turanose	1.19 *	1.13	1.36 *	1.25 *
Galactose	1.44 *	1.59 *	1.09	1.17
Fructose	1.29 *	1.36 *	1.02	1.08
Glucose	1.4 *	1.51 *	1.06	1.14
Sucrose	1.21 *	1.09	1.07	1.19
4-Keto-glucose	1.59 *	1.09	1.43 *	0.84
Arabinose	1.36 *	0.71 *	1.23 *	0.18 *
Mannose	1.06	1.11	0.88	0.73 *
Xylose	0.82 *	1.37 *	0.82 *	0.98
Glucopyranose	0.81 *	2.09 *	0.84	1.41 *
**Organic acids**	**2 h**	**1 d**	**6 d**	**32 d**
Pentonic acid	1.77 *	0.78 *	1.04	1.46 *
Succinic acid	1.13	1.2 *	1.26 *	1.54 *
Hydroxypyruvic acid	1.16	1.07	1.28 *	0.71 *
2-Ketoglutaric acid	1.06	1.1	0.65 *	1.31 *
2-Keto-d-gluconic acid	1.05	1.56 *	1.13	2.52 *
Gluconic acid	1	1.81 *	0.92	1.04
Isocitric acid	0.85	2.1 *	1.11	1.15
2,3,4-Trihydroxybutyric acid	0.84	1.67 *	0.61 *	1.2 *
Ethanedioic acid	0.83 *	1.26 *	1.11	1.55 *
Citric acid	0.83 *	1.53 *	1.22 *	1.92 *
Malic acid	0.63 *	1	0.77 *	1.41 *
4-N-Trimethylsilylmethylaminobutyric acid	0.56 *	1.03	0.76 *	1.33 *
GABA	0.41 *	1.23 *	0.76 *	1.25 *
**Amino acids**	**2 h**	**1 d**	**6 d**	**32 d**
Ornithine	2.63 *	3.69 *	2.34 *	2.87 *
Valine	0.48 *	0.99	0.46 *	0.7 *
5-oxo-L-proline	0.45 *	1.08	0.73 *	1.14
Glycine	0.37 *	1.42 *	0.65 *	1.44 *
Alanine	0.31 *	2.22 *	0.69 *	1.25 *
Threonine	0.29 *	1.12	0.47 *	0.92
Aspartic acid	0.14 *	0.99	0.35 *	0.8 *
L-proline	0.13 *	0.99	0.19 *	0.65 *
Serine	0.13 *	1.27 *	0.2 *	0.64 *
Glutamine	0.11 *	0.86	0.3 *	0.73 *
Asparagine	0.03 *	0.34 *	0.25 *	0.89
**Alcohols**	**2 h**	**1 d**	**6 d**	**32 d**
Glycerol	1.61 *	1.2 *	0.91	2.23 *
Arabitol	1.05	1.04	0.39 *	0.92
Rhamnitol	0.98	0.97	0.76 *	1.73 *
Sorbitol	0.94	0.68 *	1.87 *	1.8 *
**Fatty acids**	**2 h**	**1 d**	**6 d**	**32 d**
Oleic acid	UP *	UP *	UP *	UP *
Tetradecanoic acid	UP *	UP *	UP *	UP *
Octadecanoic acid	0.65 *	1.11	0.94	1.66 *
9,12-Octadecadienoic acid	0.57 *	0.84	1.05	0.93
Hexadecanoic acid	0.62 *	1.06	0.99	1.38 *
Hexadecanoic acid,2,3-bisoxypropylester	0.68 *	0.39 *	0.81 *	2.18 *
**Others**	**2 h**	**1 d**	**6 d**	**32 d**
Phosphate	0.89	1.48 *	1.33 *	1.48 *

Samples at 2 h after treatment and 1, 6 and 32 d after harvest were used for the differential primary metabolic profiling analysis. A 300 mg sample was extracted in 2,700 μl of methanol. Extracts were determined with GC-MS used ribitol as internal standard. A total of 45 metabolites were differentially accumulated in heat treated pericarp. This table shows the ratio (HT/control during the same period of storage). *: significant difference (*P* <0.05). UP: metabolite was detected in the heat treated pericarp but not in the control pericarp.

GC-MS metabolite profiling (primary metabolites) was complemented using HPLC-QTOF-MS analysis of mostly secondary metabolites. Statistical filtering was applied to the mass signals data to estimate the number of up/down-regulated metabolites in heat treated pericarp. After MS/MS analysis, a total of 58 metabolites were found to be significantly regulated (2-fold; *P* < 0.05) in heat treated pericarp when compared to control pericarp during the same period of storage, and 27 of these metabolites were identified (Table 3). The metabolites detected by the HPLC-QTOF-MS technology contained mainly polyphenols and flavonoids (Table 3).

**Table 3:** Secondary metabolites specifically accumulated in heat treated pericarp

**Secondary metabolites**	**2 h**	**1 d**	**6 d**	**32 d**
Quercetin-dihexose-deoxyhexose	16.71	14.81	15.42	n.s.
Hesperetin	12.32	2.08	n.s.	n.s.
Naringenin chalcone-hexose	15.04	n.s.	4.77	n.s.
Vanillic acid	14.73	n.s.	n.s.	14.37
Hydroxybenzoic acid-hexose	14.99	n.s.	n.s.	n.s.
Diosmin	4.06	n.s.	n.s.	n.s.
Rutin	3.69	n.s.	n.s.	n.s.
Phloretin-C-diglycoside	14.97	n.s.	0.06	n.s.
Chlorogenic acid	15.96	0.06	0.12	n.s.
Isosakuranetin	n.s.	16.42	n.s.	n.s.
Qucercetin	n.s.	16.03	0.14	0.12
Neohesperidin	n.s.	1.31	0.14	n.s.
Protocatechuic acid	n.s.	0.09	n.s.	n.s.
Caffeic acid hexose	n.s.	0.06	0.20	15.90
Naringenin	n.s.	n.s.	15.31	n.s.
Caffeic acid	n.s.	n.s.	0.09	n.s.
Hydroxylated naringenin-hexose	n.s.	n.s.	0.10	n.s.
Sinensetin	n.s.	n.s.	0.08	n.s.
p-Coumaroyl quinic acid	n.s.	n.s.	0.07	n.s.
Feruloylquinic acid	n.s.	n.s.	0.06	n.s.
Pantothenic acid -hexose	n.s.	n.s.	0.06	n.s.
Quercetin-O-dihexoside	0.08	n.s.	1.13	0.46
Cinnamic acid	0.11	n.s.	n.s.	n.s.
Eriodictyol-O-dihexoside	0.06	n.s.	n.s.	0.06
Ferulic acid	0.42	n.s.	0.09	n.s.
Sinapic acid	0.05	n.s.	0.07	n.s.
Jasmonic acid	0.07	n.s.	0.06	n.s.

Samples at 2 h and 1, 6 and 32 DAT were used for the differential secondary metabolic profiling analysis using the HPLC-QTOF-MS instrument. Four replicates were compared and statistically analysed per sample using Student’s t-test; *P <* 0.05. After MS/MS analysis, a total of 58 metabolites were found to be significantly regulated (2-fold) in heat treated pericarp when compared to control pericarp (during the same period of storage), and 27 of these metabolites were identified. This table shows the ratio (HT/control during the same period of storage). n.s.: not significant.

### HT up-regulated stress response proteins in citrus pericarp

In disease response, HT induced Beta-1, 3-glucanase (H25) and Class III chitinase (H20) accumulated in pericarp (Table 1), where these proteins are involved in reducing stress resistance in plants. In the stress response, HT up-regulated 17.7 kDa heat shock protein (H22) and low molecular weight heat-shock protein (H24) accumulated in pericarp, especially at early storage stage (Table 1). Also, other proteins accumulated in heat treated pericarp in higher amounts when compared to control pericarp during the same period of storage, which might be indirectly involved in the fruit’s response to stress, including the pyruvate dehydrogenase E1 beta subunit isoform 2 (H7), chloroplast stromal ascorbate peroxidase (H18), putative ATP synthase beta subunit (H36), putative inorganic pyrophosphatase, isoflavone reductase related protein (H10), and cytoplasmic malate dehydrogenase (H4, Table 1).

### HT down-regulated reactive oxygen species metabolism-related proteins in citrus pericarp

HT down-regulated reactive oxygen species metabolism-related proteins, especially during later periods of storage (Table 1), including oxidoreductase, zinc-binding dehydrogenase family protein (H11), putative NAD(P)H oxidoreductase, isoflavone reductase (H12), aldo/keto reductase family protein (H13), copper/zinc superoxide dismutase (H16), and putative NAD(P)H oxidoreductase, isoflavone reductase (H17). Also, other proteins were down-regulated in heat treated pericarp (relative to control), such as triosephosphate isomerase, cytosolic (H5), abscisic stress ripening-like protein (H19), and thiazole biosynthetic enzyme, chloroplast precursor (H30). In addition, some proteins were up-regulated in heat treated pericarp (relative to control) only in the middle of the experiment, including, phosphoglycerate kinase, putative (H2), pyruvate dehydrogenase E1 beta subunit isoform 2 (H7), aldo/keto reductase family protein (H13), mitochondrial processing peptidase (H15), putative inorganic pyrophosphatase (H29). Putative ATP synthase beta subunit (H36) was up-regulated at the end but down-regulated at the beginning in heat treated pericarps (relative to control).

### HT increased contents of sugars and flavonoids in citrus pericarp

Primary metabolite profiling of the HT and control pericarp revealed prominent changes in a group of metabolites during the entire storage phases (Table 2), mainly including sugars and fatty acids. Interestingly, the contents of many kinds of sugars were increased in heat treated pericarp (relative to control) at 2 h after HT, but decreased to the same level with control at later storage phases, such as galactose, fructose, glucose, sucrose and 4-keto-glucose. Some primary metabolites contents were increased in heat treated pericarp (relative to control) during whole storage, including oleic acid, tetradecanoic acid and ornithine (Table 2).

Secondary metabolite profiling of the HT and control pericarp revealed that a group of metabolites were increased in heat treated pericarp (relative to control) during some periods of storage, including quercetin-dihexose-deoxyhexose, hesperetin, hydroxybenzoic acid-hexose, naringenin, naringenin chalcone-hexose, vanillic acid, diosmin, rutin and isosakuranetin, which all might play a vital role in the response to stress.

### HT decreased contents of organic acids, amino acids and phenylpropanoids

Primary metabolic profiling revealed organic acids, fatty acids and amino acids were down-regulated in heat treated pericarp when compared to control pericarp, especially at 2 h after heat treatment. Levels of five types of organic acids (citric acid, malic acid, 4-N-Trimethylsilylmethylaminobutyric acid, oxalic acid and GABA) had decreased in heat treated pericarp compared to the control at 2 h after HT (Table 2), but levels of ten types of organic acid increased in heat treated pericarp (relative to the control) at 32 DAT (Table 2). The content of ten types of amino acids were lower in heat treated pericarp than in the control at 2 h after HT, while only five of them were lower in heat treated pericarp than in the control at 32 DAT, although two of them were higher in heat treated pericarp than in the control at 32 DAT (Table 2). In a similar way, compared to organic acids, levels of four types of fatty acids decreased in heat treated pericarp compared to the control at 2 h after heat treatment; levels of three of them increased in heat treated pericarp compared to the control at 32 DAT (Table 2). There is common occurrence here; levels of many types of organic acids, amino acids and fatty acids decreased in heat treated pericarp compared to the control at 2 h after HT, but levels of many of them increased in heat treated pericarp compared to the control at 32 DAT. Compared to increased sugar concentrations, it appears there is a sudden transformation of organic, amino and fatty acids into sugars which is driven by HT; also, levels of many types of organic, amino and fatty acids increased in heat treated pericarp compared to the control during the later stages of storage.

Further metabolic characterization of chemicals which induced resistance in heat treated pericarp was performed using an HPLC-QTOF-MS. Contents of ferulic acid, sinapic acid, cinnamic acid and caffeic acid, the precursors of lignin synthesis, decreased in heat treated pericarp when compared to the control pericarp, especially at 2 h and 12 DAT. Moreover, the contents of other secondary metabolites decreased in heat treated pericarp, especially at 2 h or 12 DAT, such as hydroxylated naringenin-hexose, sinensetin, p-coumaroyl quinic acid, feruloylquinic acid, pantothenic acid-hexose, quercetin-O-dihexoside, eriodictyol-O-dihexoside, and jasmonic acid.

### HT decreased H_2_O_2_ content in citrus pericarp

**Figure 4:**
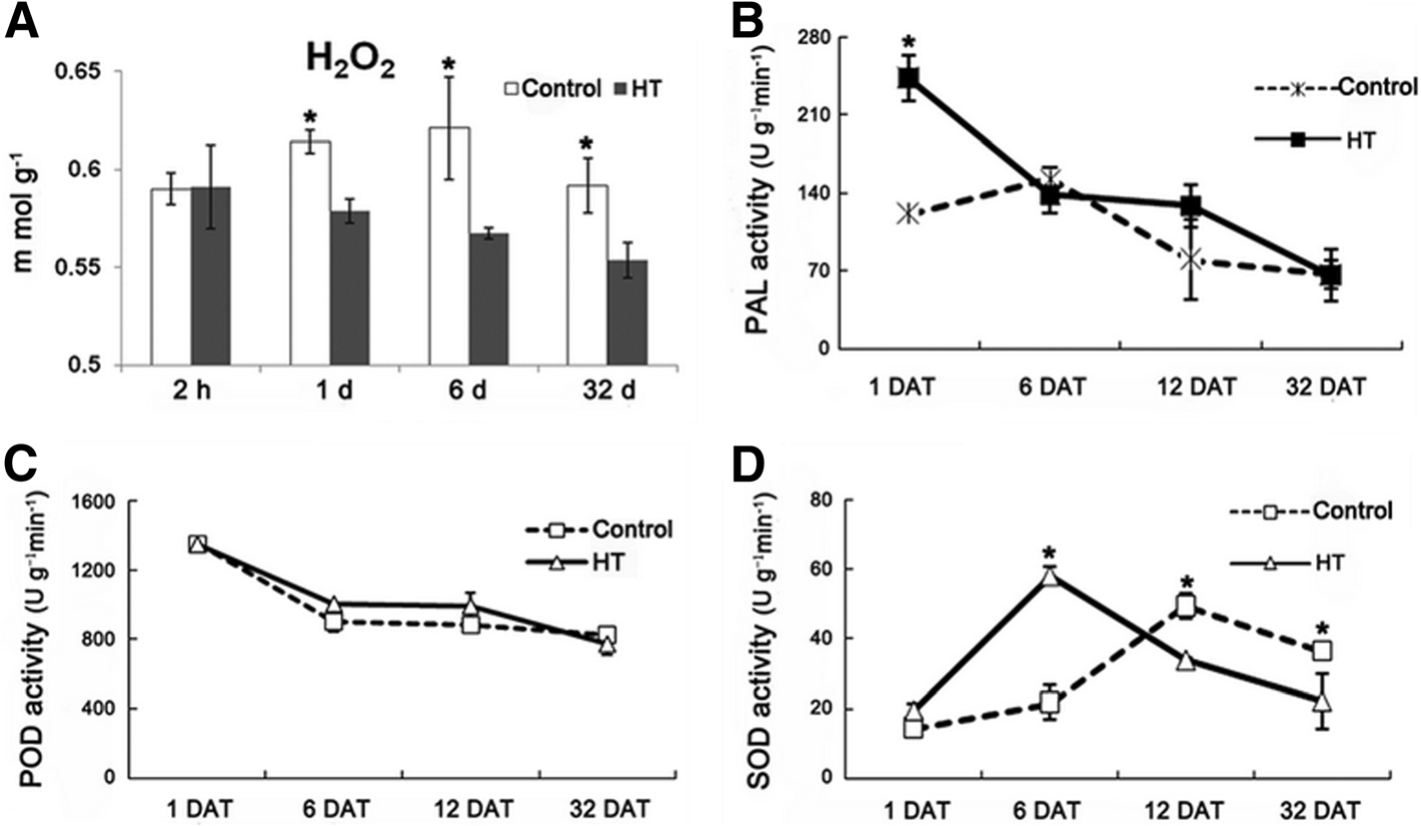
**Changes of H**_**2**_**O**_**2 **_**content and enzyme activity in HT and control pericarp. A**: H_2_O_2_ content. Samples at 2 h and 1, 6 and 32 DAT were used for H_2_O_2_ content determined in HT and control pericarp. **B-D**: Enzyme activity. Samples at 1, 6, 12 and 32 DAT were used for enzyme activity determination. PAL: phenylalanine ammonia-lyase; SOD: superoxide dismutase; POD: peroxidase. Four replicates were completed and statistically significant differences analysis was conducted between HT and control pericarp during the same period using Student’s t-test. *: significant difference (*P* < 0.05). Mean values and SE bars are provided.

The content of H_2_O_2_ decreased in heat treated pericarp during the storage phase while the content of H_2_O_2_ increased in the control pericarp (Figure 4A). This suggests HT decreased the content of H_2_O_2_ in pericarp, and the content of H_2_O_2_ was held at a lower level in heat treated pericarp when compared to control pericarp during storage.

Superoxide dismutase (SOD) and peroxidase (POD) play important roles in deoxidization of H_2_O_2_. In this study, the activity of POD showed no significant change between HT and the control (Figure 4C). The activity of SOD was higher in heat treated pericarp relative to the control at 6 DAT, but was lower in heat treated pericarp relative to the control at 12 and 32 DAT (Figure 4D). So, the activity of SOD fluctuated during the entire storage period in heat treated pericarp compared to the control (Figure 4D).

Concerning redox regulation, most of the proteins were down-regulated in heat treated pericarp when compared to control pericarp, especially in the later stages of storage (Table 1). This suggests the redox reaction was weaker in heat treated pericarp during storage.

### HT increased fruit firmness and the content of lignin in citrus pericarp

**Figure 5:**
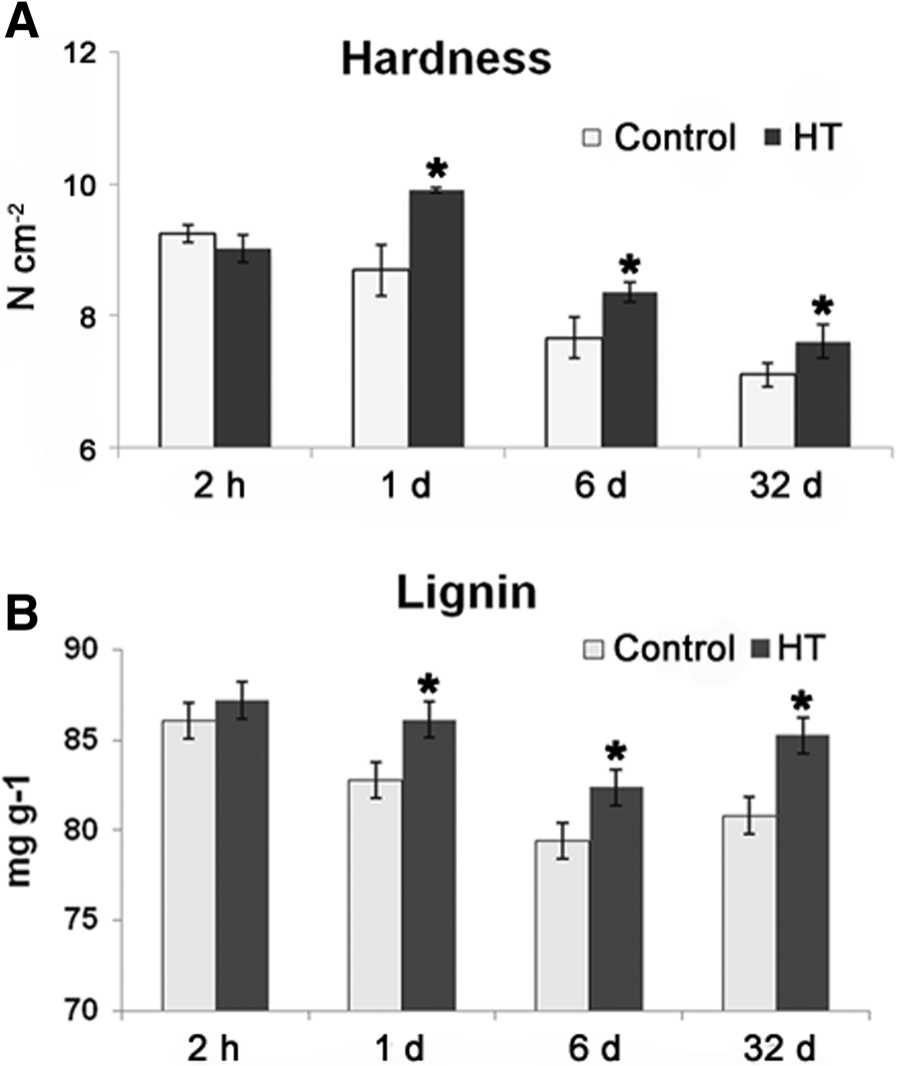
**Changes of fruit hardness (A) and lignin content (B) in HT and control pericarp.** Samples at 2 h and 1, 6 and 32 DAT were used for fruit hardness and lignin content determination. An analysis of statistically significant differences was conducted between HT and control pericarp during the same period using Student’s t-test. *: significant difference (*P* < 0.05). Mean values and SE bars are provided.

Fruit hardness gradually decreased during storage (Figure 5A); however, HT increased fruit hardness, especially at 1 DAT (Figure 5A), which led to the heat treated fruit remaining firmer than the control fruits during the entire storage period. Lignin is an important component responsible for fruit hardness. So, samples from 2 h after treatment and 1, 6 and 32 DAT were used to detect lignin content changes over time. Results show HT significantly induced lignin accumulation in pericarp (Figure 5B). Also, the activity of phenylalanine ammonia-lyase (PAL), a critical enzyme in lignin synthesis, initially increased in heat treated pericarp at 1 DAT but subsequently fell into the same level as in the control (Figure 4B). Furthermore, levels of ferulic acid, sinapic acid, cinnamic acid and caffeic acid, the precursors of lignin synthesis, declined in heat treated pericarp when compared to control pericarp (Table 3). This suggests a rapid transformation occurred from ferulic acid, sinapic acid, cinnamic acid and caffeic acid to lignin in heat treated pericarp after HT.

## Discussion

### HT improved fruit pathogen resistance without negatively impacting fruit quality

Physical treatments are used frequently to keep fruit fresh, including microporous membranes, pre-storage treatment (sweating), ultraviolet illumination, radiofrequency treatment, heat treatments (heat therapy), and other storage techniques. Postharvest heat treatments such as hot water treatment and hot air treatment provide a type of quarantine, inhibit the development of pathogens, enhance fruit resistance to chilling injury in cold-sensitive cultivars, and help preserve fruit quality during storage and extend its shelf life [7]. In the present study, HT effectively inhibited the development of pathogens. Also, HT (52°C, 2 min) caused a statistically significant reduction the incidence of chilling injury [9,11].Also, postharvest HT had no obvious negative effects on fruit flesh quality during subsequent storage. This suggests HT increased the ability of fruit to tolerate storage stress without affecting their commercial qualities, and shows strong potential application in the citrus industry.

### HT induced stress response proteins

HT can effectively inhibit fruit chilling injury during cold storage [12]. After HT, some proteins were up-regulated, while others were down-regulated. The up-regulated proteins might be responsible for heat induced fruit resistance. Interesting, some proteins were up-regulated at 1 d after HT, and decreased to the previous level of control during the storage period. Those kinds of proteins might be just involved in response to HT, but not play a vital role in fruit disease resistance. The proteins, which are up-regulated during whole storage period might be directly involved in fruit disease resistance.

In the present study, heat shock protein (HSP) 17.7, putative HSP 70 and low molecular weight HSP were up-regulated in heat treated pericarp compared to the control during entire storage period (Table 1). HSPs are encoded by a multigene family and are located in different subcellular compartments [13]. These molecular chaperones play a crucial role in protecting plants against stress by re-establishing normal protein configuration and thus helping to maintain cellular homeostasis [14]. Abiotic stresses usually causes protein dysfunction; so maintaining proteins in their functional configurations and preventing the aggregation of non-native proteins is particularly important for cell survival under stress [14]. HSPs are responsible for protein folding, assembly, translocation and degradation during cellular processes. HSPs also stabilize proteins and membranes, and can assist in protein refolding under stressful conditions [15]. Our results suggest HSPs play a crucial role in reducing the susceptibility of HT fruit to biotic and abiotic stress.

HT significantly inhibited pathogen development and fruit decay during storage [16]. Chitinase and β-1, 3-glucanase were up-regulated in heat treated fruit in this study; these enzymes have long been thought to be active in fruit antifungal defences. Pathogenesis-related (PR) proteins have been classified into five groups and two of these groups contain proteins with β-1, 3-glucanase and chitinase [17]. Chitinase catalyses the hydrolysis of chitin, a β-1, 4-linked polymer of N-acetyl D-glucosamine and is a major component of cell walls of most phytopathogenic fungi [18]. Crude protein extracts of chitinase and β-1, 3-glucanase were observed to effectively inhibit fungal activity [19]. Also, β-1, 3-glucanase and chitinase are thought to contribute to the level of resistance provided and are known to inhibit mycelial growth of a wide range of fungal pathogens [20]. This suggests β-1, 3-glucanase and chitinase were directly involved in HT induced pathogen resistance in fruits.

### HT increased the content of stress response metabolites

Primary metabolites are directly involved in normal growth, development, and reproduction. In the present study, the content of ornithine, oleic acid and tetradecanoic acid increased in heat treated pericarp compared to the control during storage, and levels of seven types of sugars increased in heat treated pericarp compared to the control at 2 h after HT (Table 2). In plants, ornithine is required for the synthesis of polyamines and alkaloids, which contribute to oxidative stress tolerance in plants subjected to severe water stress [21].

Flavonoids are important defensive compounds which protect fruit against the development of pathogens, acting either as phytoanticipins or phytoalexins [22]. Flavonoids were observed to be up-regulated in heat treated pericarp (compared to the control), including quercetin-dihexose-deoxyhexose, hesperetin, naringenin, naringenin chalcone-hexose, hydroxybenzoic acid-hexose, isosakuranetin, diosmin, and rutin, which may play a vital role in response to stress [23]. Rutin is a bioflavonoid with strong antioxidant activity, which had an antimicrobial effect on pathogens, as evidenced by reduced conidial germination and appressorium formation of the pathogen [22]. Quercetin protected cells from oxidative stress-induced cell death by blocking both the cyclooxygenase and lipooxygenase pathways [24]. Quercetin has a protective function against oxidative stress by reducing oxidative injury to the cells during H_2_O_2_ treatment [25]. Also, plant extracts rich in quercetin have been applied as an interesting resource for functional food products and other foods used to reduce the risks of age-related macular degeneration [25]. Hesperidin acts as an antioxidant, based on in vitro studies, and hesperidin also provides strong cellular antioxidant protection against the damaging effects induced by stress [26]. Diosmin, hesperidin and naringin are flavonoid glycosides that occur naturally in citrus fruits; they exert a variety of properties such as antioxidant and free radical scavenging [27]. Interestingly, naringenin and rutin are not only involved in response to stress, but also were assumed to be signalling molecules [23].

### HT induced fruit pathogen resistance and increased lignin content

The functions of phenylpropanoid compounds in plant defence range from preformed or inducible physical and chemical barriers against infection [10]. Defensive functions are not restricted to a particular class of phenylpropanoid compounds, but are found in the lignification of monolignols to lignin [28]. In the present study, HT decreased the level of precursors needed in lignin synthesis (ferulic acid, sinapic acid, cinnamic acid and caffeic acid), but increased lignin content (Table 3, Figure 5). Lignin is one of the major components of secondary cell walls, providing cells with mechanical support and isolation from the environment. In plants, cells can sense cell wall polymer structure and this activates trans-plasma membrane sensor proteins which might trigger a cascade of signal transduction events interwoven with other pathways that might modulate cellular functions by the activation or inhibition of specific transcription factors as well as by affecting posttranslational control of gene expression and protein function [29]. If a cell wall is damaged, cells activate a series of responses to the invading disease organisms, which might negatively impact fruit quality. The accumulation of lignin induced by HT creates a thickening of cell walls which forms an effective physical barrier to pathogens, delaying the invasion of disease organisms. Also, HT increased fruit firmness; this might be a result of HT induced lignin thickened fruit cell walls.

### HT reduced the content of H_2_O_2_

HT is an effective method for improving citrus storability. In storage, fruits suffer a variety of biotic and abiotic environmental stresses. All these stresses could lead to the generation of reactive oxygen species (ROS). To avoid the accumulation of ROS, which may lead to cell death, plants have up-regulated enzymatic and non-enzymatic antioxidants to scavenge ROS and alleviate their negative effects [30]. In this study, HT down-regulated H_2_O_2_ content in pericarp, which was consistent with the effect of HT on yeast cells. HT yeast cells accumulated less ROS than untreated cells in response to stresses [31].

Also, isoflavone reductase (IFR) might have an important role as an antioxidant [32]. IFR is an enzyme involved in the production of isoflavone phytoalexins and it accumulates in response to pathogenic attack, fungal elicitor and biotic stress [33]. Isoflavonoids are important secondary metabolites for essential physiological processes. Kim *et al.*[32] reported the isoflavone reductase-like gene may act as a down-regulator by producing antioxidant chemicals to prevent the elevation of ROS. In this study, isoflavone reductase, a related protein (H10), was up-regulated in heat treated pericarp when compared to control pericarp (Table 1), which might be another factor leading to lower H_2_O_2_ levels and stronger resistance to pathogens in heat treated pericarp when compared to control pericarp.

Ascorbate peroxidase scavenges H_2_O_2_ in specific organelles of the cell [34]. Also, Cu/Zn-SOD is the main cellular isoform of SODs which play a key role in the antioxidant defence system through the disproportionation of H_2_O_2_[34]. Both of these can prevent ROS caused damages to the cellular membrane and act as a primary defence mechanism when organisms were exposed to oxidative stress. These proteins are considered the main enzymatic and non-enzymatic systems for protecting cells against oxidative damage, and are responsible for disease resistance. In response to biotic stress, these proteins also act as the primary defence mechanism against oxidative stresses caused by pathogens, therefore preventing damage of ROS to cellular membranes. However, the activities of POD and SOD were not elevated in Heat treated pericarp when compared to control pericarp, which might be caused by the lower H_2_O_2_ content in heat treated pericarp. Also, other proteins involved in scavenging ROS were down-regulated in heat treated pericarp when compared to control pericarp, including oxidoreductase (H11), oxidoreductase (H12, 17), aldo/keto reductase (H13), 2-oxoacid dehydrogenase (H14), superoxide dismutase (H16). This suggests HT induced a level of resistance to pathogens attacking fruits by causing a decline in fruit H_2_O_2_ content and by down-regulated scavenging ROS enzymes.

### Role of sugars, organic acids and amino acids in improving storability

In this study, the profiling of primary metabolics showed HT induced the accumulation of sugars at early stages of storage (Table 2). Soluble sugars, especially sucrose, glucose, and fructose, accumulated when fruits were subjected to stress, and accumulated soluble sugars play an obviously vital role in the response of fruit to a number of stressors [35]. Soluble sugars do not only act as nutrients and metabolites, but also act as signal substance in the modification of redox homeostasis [36]. However, less information was available on how other sugars are involved in the stress response, especially arabinose, mannose, galactose, and ribose.

Moreover, primary metabolic data shows HT reduced organic acids and amino acids content especially at 2 h after treatment in this study. Although organic acids and amino acids play an obvious central role in energy and metabolism at the cellular and whole-organism levels, there seems to be a rapid conversion from organic acids and amino acids to sugars in heat treated pericarp. However, little is known about the decreased levels of organic acids and amino acids seen during a stress response, and further study will be required to validate this response.

## Conclusions

This study provides a broad picture of differential accumulation of proteins and metabolites, and gives a new insight into how HT increases fruit stress resistance during subsequent storage of citrus fruit. In metabolic substrates, the up-accumulation of secondary metabolites played vital roles in increasing the ability of fruit to deal with stress. Flavonoids are directly involved in the response to external stress. The increased lignin and decreased H_2_O_2_ contents involved in HT probably increased fruit resistibility in response to external stress.

## Methods

### Sample collection

‘Kamei’ Satsuma mandarin (*Citrus unshiu* Marc.) fruits were harvested at commercial maturity (fruit colour has turned completely orange and the level of soluble solids approaches 9%) from an orchard in the city of Yichang, Hubei Province, China, in October 2009. Fruits of a uniform size and colour, free of visible injury or blemishes, were selected for the experiments. Three hundred kilograms of fruit were divided into two equal parts, 150 kg were used for the HT and the other 150 kg used for a control. After treatments, fruits were stored in a ventilated warehouse (storage temperature: 12–16°C; relative humidity: 90–95%). Samples were collected at 2 h, and 1, 2, 3, 4, 6, 9, 12, 16, 20, 24, 28, 32, 38, 44, 50 and 60 DAT. The pericarp was sampled along the equatorial plane of each fruit; 30 fruits were selected as one sample and ground to powder in liquid nitrogen, then stored at –80°C for further analysis.

### Heat treatment (HT)

Heat treated fruits were dipped in a 52°C warm water bath for 2 min, and control fruits were dipped in a 25°C water bath for 2 min. After treatments, fruits were air dried and stored in a ventilated warehouse prior to sampling.

### Fruit quality determination

Fruits were checked for weight loss (%), respiration rate (mg kg^–1^ h^–1^) and total soluble solids (TSS,%) at 1, 2, 3, 4, 6, 9, 12, 16, 20, 24, 28, 32, 38, 44, 50 and 60 DAT. The weight was measured using an electronic hydrostatic balance (Model: MP31001, Shanghai Selon Scientific Instrument, Co., Ltd, Shanghai, China) with an accuracy of ± 0.01 g. The respiration rate of fruit was measured with an infrared gas analyser (Model: GXH-305H, Junfang Science & Technology Institute of Physical and Chemical Research, Beijing, China). TSS were determined with a refractometer (Model: Pocket PAL-1, Atago Inc., Toyko, Japan) according to the manufacturer’s instructions.

### Antifungal assay of HT in wounded citrus fruits

Blue mould (*Penicillium italicum*) was chosen as an indicator to evaluate the resistance of treated fruit to fungal infections. 150 fruits from each treatment were used for fungal inoculation. A uniform lesion (3 mm deep, 4 mm wide) was made at the equator of the fruit using a sterile nail. Aliquots of 10 μl suspension of *P. italicum* at 1×10^5^ spore ml^–1^ were inoculated into each wound site. After fungal inoculation, fruits were stored in a storage chamber (relative humidity, 95%; temperature, 25°C), and disease incidence was detected at 1, 2, 3, 4, 5, 6 and 7 d after inoculation. Disease incidence rates and lesion diameters were recorded based on the following equations:

$$\begin{array}{l} \text{Disease}\;\text{incidence}\;\text{rate}\;\left(\%\right)\\ \;=\sum \text{Number}\;\text{of}\;\text{diseased}/\text{decaying}\;\text{citrus}\\ \;/\text{total}\;\text{number}\;\text{of}\;\text{fruit}\;\text{in}\;\text{the}\;\text{treatment}\times 100; \end{array}$$

$$\begin{array}{l} \text{Lesion}\;\text{diameter}\left(\text{cm}\right)\\ \;=\sum \text{Lesion}\;\text{diameter}\;\text{in}\;\text{diseased}/\\ \;/\text{total}\;\text{number}\;\text{of}\;\text{fruit}\;\text{in}\;\text{the}\;\text{treatment} \end{array}$$

### Two-dimensional gel electrophoresis and MAILDI-TOF/TOF

Samples at 1, 6, 12 and 32 DAT were used for total protein extraction based on the method described by Isaacson *et al.*[37]. The precipitate was then nitrogen gas-dried and solubilized in the lysis buffer containing 8 M urea, 0.2% (w/v) Bio-Lyte, 4% CHAPS, 65 mM DTT. The protein concentration was determined with a Bio-Rad protein assay kit (Bio-Rad Laboratories Inc., Hercules, CA, USA) based on the Bradford method using BSA as the standard. Two-dimensional gel electrophoresis and gel staining was carried out based on the method of Yun *et al.*[38]. The stained gels were imaged with a UVP imager (Bio-Rad) in the ‘trans white’ mode using PDQuest 2-D analysis software version 7.4 (Bio-Rad). Protein spots matching and differential protein spots analysis were completed based on the method of Yun *et al.*[38]. Isoelectric points and molecular weights of the proteins were determined by comparison with the markers.

In-gel digestion and protein identification were performed using a 4800 Proteomics Analyzer MALDI TOF/TOF (Applied Biosystems, Foster City, CA) as described by Yun *et al.*[38]. MS/MS data were then submitted to the MASCOT program for protein identification against the green plants database. The following are the search parameters: trypsin enzyme, one missed cleavage, fixed modifications of carbamidomethyl (C), fragment mass tolerance of ± 0.5 Da, variable modifications of oxidation (Met), peptide tolerance of 100 ppm, and peptide charge of 1+. Only results of peptides with MS/MS *P* < 0.05 confidence were accepted.

### The primary metabolic profiling

Samples collected at 2 h and 1, 6 and 32 DAT were used for the differential primary metabolic profiling analysis. A 300 mg sample was extracted in 2,700 μl of methanol as described by Roessner-Tunali *et al.*[39]. 300 μl of 0.2 mg ml^–1^ ribitol in water was added as a quantification internal standard. A derivatization reaction was performed based on the protocol of Zhang *et al.*[40] with little modification. Extracts were incubated in 50 μl of 20 mg ml^–1^ methoxyamine hydrochloride in pyridine for 30 min at 50°C, followed by a 40 min treatment at 60°C using 50 μl BSTFA (containing 1% TMCS). Each sample (1 μl) was injected into the gas chromatograph system through a fused-silica capillary column (30 m × 0.25 mm i.d., 0.25 μm) DB-5 MS stationary phase. The injector temperature was 250°C, with a carrier gas flow rate of 1.0 ml min^–1^. The column temperature was held at 100°C for 1 min; increased to 184°C at a rate of 3°C min^–1^, increased to 190°C at a rate of 0.5°C min^–1^, increased to 280°C at 15°C min^–1^. The flow rate of the carrier helium (99.999%) gas was 1 ml min^–1^. The following were MS operating parameters: ionization voltage, 70 eV (electron impact ionization); ion source temperature, 200°C; interface temperature, 250°C. TIC (total ioncurrent) spectra were recorded in the mass range of 45–600 atomic mass units in scanning mode.

### The secondary metabolic profiling

Samples at 2 h and 1, 6 and 32 DAT were used for differential secondary metabolic profiling analysis using HPLC-MS. 300 mg dried powder was extracted with methanol, the mixture was filtered through a 0.22–mm polytetrafluoroethylene membrane filter.

Metabolite profiling was performed using a QTOF 6520 mass spectrometer (Agilent Technologies, Palo Alto, CA, USA) coupled to a 1200 series Rapid Resolution HPLC system as described by Page *et al.*[41]. 2 μl of sample extract was loaded onto a Zorbax Eclipse Plus C18 1.8 μm, 2.1 × 100 mm reverse-phase analytical column (Agilent Technologies). Mobile phase A comprised 0.1% formic acid in water and mobile phase B was acetonitrile. The following gradient was used: 0 min–10% B; 20 min–95% B; 22 min–95% B; 22.1 min–10% B; 30 min–10% B. The flow rate was 0.3 ml min^–1^ and the column temperature was held at 35°C for the duration. The source conditions for electrospray ionization were as follows: gas temperature was 350°C with a drying gas flow rate of 10 l min^–1^ and a nebulizer pressure of 40 psig. The capillary voltage was 3.5 kV in both positive and negative ion mode. The fragmentor voltage was 135 V and the skimmer was 65 V.

### Assay of phenylalanine ammonia-lyase (PAL), superoxide dismutase (SOD) and peroxidase (POD) activities

Samples collected at 1, 6, 12 and 32 DAT were used for enzyme activity determination. A 5 g sample was homogenized in 25 ml of 0.05 M sodium borate buffer (pH 8.8 for PAL, containing 5 mM β-mercaptoethanol) and 25 ml of ice-cold 100 mM sodium phosphate (pH 7.8 for POD and SOD, containing 0.5 g polyvinyl polypyrrolidone). PAL activity was determined by measuring the absorbance of cinnamic acid at 290 nm over a period of 30 min at 30°C [42]. POD activity was analysed using guaiacol as the substrate [43]. SOD activity was determined using the xanthine-xanthine oxidase method, and nitro blue tetrazolium was used as the indicator of superoxide radical production [44].

### Determination the content of H_2_O_2_ in the peel

Samples collected at 2 h and 1, 6 and 32 DAT were used to measure hydrogen peroxide levels based on the methods of Patrick and Wagner [45] with some modifications. Briefly, peel tissues (0.5 g) were ground in liquid nitrogen and homogenized in 5 ml of physiological saline. Samples were then subjected to an ultrasonic bath for 15 min and centrifuged at 6,000 g for 20 min. The supernatants (50 μl) were subsequently added into 0.5 ml of solution I and warmed in a 37°C water bath for 10 min before 0.5 ml of solution II was added. The absorbance of the mixture was measured at 405 nm and the content of H_2_O_2_ in each sample was calculated by comparison with a standard calibration curve.

### Determination of fruit firmness

Samples collected at 2 h and 1, 6 and 32 DAT were used for the determination of fruit firmness. Eighteen fruits were measured and every fruit was measured on three different spots around the equatorial plane. Firmness was measured with a fruit sclerometer (GY-B, Jilin, China) with 4 mm diameter and expressed as N cm^–2^ and the average value per fruit was used for further analysis, after removing three largest and three smallest measurements; a total twelve replicates were comparative statistically analysed per sample using Student’s t-test (*P* < 0.05).

### Determination of lignin in pericarp

Samples collected at 2 h and 1, 6 and 32 DAT were used to measure fruit lignin content. Lignin levels were determined using the method described by Lei *et al.*[46].

### Statistical analysis

The experimental design was completely randomized with more than three replications. Four individual replicates were completed in 2-DE, GC-MS, HPLC-MS, H_2_O_2_ content, lignin content and enzyme activity determination. Twelve replicates were completed to measure weight loss, respiration rate, total soluble solids and fruit firmness. Data for each sample was statistically analysed using Student’s t-test (*P* < 0.05).

## Abbreviations

2-DE: Two-dimensional gel electrophoresis; GC-MS: Gas chromatography coupled to mass spectrometry; HSP: Heat shock protein; HT: Heat treatment; IFR: Isoflavone reductase; LC-QTOF-MS: Liquid chromatography quadrupole time-of-flight mass spectrometry; MALDI-TOF MS: Matrix-assisted laser desorption/ionization-time-of-flight tandem mass spectrometry; NCBI: National center for biotechnology information; PAL: Phenylalanine ammonia-lyase; POD: Peroxidise; PR: Pathogenesis-related; ROS: Reactive oxygen species; SOD: Superoxide dismutase; TSS: Total soluble solids.

## Competing interests

The authors declare that they have no competing interests.

## Authors’ contributions

ZY, HJG and YJC conceived the study, designed all the experiments and performed silicon and biochemical analyses. SZL carried out primary metabolite detection and statistical analysis. SJ carried out secondary metabolite detection and statistical analysis. ZY, HJG, TL, QX, JX, YJC and XXD interpreted the experimental dada and participated in writing the manuscript. All the authors read and approved the final manuscript.
